# JAHEE internal evaluation

**DOI:** 10.1093/eurpub/ckac129.434

**Published:** 2022-10-25

**Authors:** A Vantarakis

**Affiliations:** Department of Public Health Medical School, University of Patras, Athene, Greece

## Abstract

The evaluation of the project for both the intermediate and the final report was based on an internal evaluation conducted by Work Package on Evaluation and on an external evaluation conducted by the Center for Global Health Inequality Research (CHAIN). The project evaluation as based on systematic and continuous monitoring of processes, outputs and outcomes indicators aimed to guarantee the achievement of the planned objectives and the identification of the lessons learned for future health inequalities programs. The main results will be presented.

